# Reproduction performance and blood biochemical parameters in dairy cows: Relationship with oxidative stress status

**DOI:** 10.14202/vetworld.2018.883-888

**Published:** 2018-06-30

**Authors:** Sofiane Boudjellaba, Lynda Ainouz, Safia Tennah, Soraya Temim, Mokrane Iguer-Ouada

**Affiliations:** 1 Laboratoire de Recherche Gestion des Ressources Animales Locales. École Nationale Supérieure Vétérinaire d’Alger, Algeria; 2 Laboratoire de Biologie et Physiologie Animale, ENS, Kouba, Alger. École Nationale Supérieure Vétérinaire d’Alger, Algeria; 3 Laboratoire de Recherche Santé et Production Animales. École Nationale Supérieure Vétérinaire d’Alger, Algeria; 4 Laboratoire Associé en écosystèmes Marins et Aquacoles, Faculté des Sciences de la Nature et de la Vie, Université de Béjaia, Bejaia 06000, Algeria

**Keywords:** cow, glutathione S-transferase, malondialdehyde, reproduction performance

## Abstract

**Background and Aim:**

During the last decades, reproduction performances declined dramatically worldwide, but little is known concerning the involvement of oxidative stress as a causative factor. Oxidative stress may act at different levels, with negative impacts on cell membrane integrity and other active molecules with potential subsequent effects on reproduction. The aim of the current study was to investigate the oxidative stress status in cows according to their reproductive performances.

**Materials and Methods:**

Peripheral blood concentration of two oxidative stress biomarkers, glutathione S-transferase (GST) and malondialdehyde (MDA), and other biochemical parameters (glucose, total lipids, cholesterol, triglycerides, albumin, total proteins, calcium, urea, creatinine, direct bilirubin, alanine aminotransferase, aspartate aminotransferase, and alkaline phosphatase) were determined in 40 healthy cows. Body condition score (BCS), calving to first service interval (FSI), calving to conception interval (CCI), and the number of service per conception (SPC) were simultaneously recorded for each cow.

**Results:**

Concerning FSI, three groups were established: Group 1 (from 44 to 60 days), Group 2 (from 60 to 70 days), and Group 3 (from 70 to 80 days). For CCI, two groups were considered: Group 1 (<110 days) and Group 2 (>110 days). MDA showed significant high values only in cows with the lowest BCS (1.5) compared to cows with BCS note of 2.5 and 3.5. No significant difference was observed in cows oxidative stress status (MDA and GST) according to reproductive performances (FSI, CCI, and SPC) in all studied groups.

**Conclusion:**

The results revealed relatively altered oxidative stress status in cows with abnormal reproductive performances; however, no significant difference was recorded whatever the considered reproductive parameter.

## Introduction

Oxidative stress has been termed as the unbalance between oxidants (reactive oxygen species [ROS] or free radicals) and antioxidants defenses in favor of oxidants, which may lead to tissue injury. Free radicals are any chemical species that contain unpaired electrons and considers as inherently unstable and highly reactive molecules [1]. Particularly, the adverse effects of oxidative stress to reproduction system involve damage to oocyte DNA, ovary, and endometrium with consequent impacts on fertility outputs [2]. Overall, reports suggested a role of oxidative stress in the etiologies of dairy cattle disorders and showed that supplementation with active antioxidants could ameliorate metabolic and infectious diseases [3]. It is established a dynamic relationship between oxidant and antioxidant status during estrus cycle in healthy cows, and it is speculated that oxidative stress has a crucial physiological role in facilitating the ovulation process in estrus synchronized dairy cows [4]. In addition, a relationship between the physiological status associated with parturition and the breakdown in overall antioxidant potential is established both in humans and dairy cow [5,6]. The impact of oxidative stress during the transition period may be a major underlying factor of inflammatory and immune dysfunction in dairy cattle as supported by *in vivo* and *in vitro* studies [7]. Internationally, reproductive performance of dairy cattle is declining, and it is well recognized that high milk production, low body condition, energy deficiency, disease, inbreeding, and management failures affect reproduction negatively [8]. Lucy, 2001, confirmed that the reproductive physiology of dairy cows has changed over the past 50 years [9]. Consequently, management practices during periparturient period have been the focus of different research groups offering several clinical and biochemical markers as indicators of metabolic and health disorders [10].

Concerning reproduction, different parameters are commonly used to measure cow’s performances as the interval from calving to first service (CFS) and the interval from calving to conception (CCI). Similarly, it is well known that several factors related to the animal itself or its environmental impact significantly reproductive performances; however, little research has paid particular attention to the relationship between oxidative stress and reproductive performances.

Therefore, the objective of our study was to demonstrate whether reproductive performances were affected by cows oxidative stress status throughout the peripartum period. The experimental design consisted of studying the relationship between calving to first service interval (FSI) and CCI (mean±standard error [SE]), number of service per conception (SPC) (mean±SE), and cows BCS on one side and two oxidative stress biomarkers glutathione-S-transferase (GST) and malondialdehyde (MDA) on the other side.

## Materials and Methods

### Ethical approval

The experiment was carried out in accordance with the guidelines laid down by the Directive 2010/63/EU of the European Parliament for Animal Ethics Committee for the use of animal experimentation.

### Animal’s experimental location

The study was carried out on 40 dairy cows (Holstein Friesian, French Montbeliard, and Brown Swiss cows) in the Technical Livestock Institute (iTELV) located in Baba Ali, Algiers (Algeria). All animals were kept under identical conditions of feeding and reproductive management. The cows were tied to stalls and exercise was allowed in a large paddock. Grass, hay, and concentrate were fed every day, and milking was realized twice a day. The voluntary waiting period was 40 days. To evaluate reproductive performance, we recorded calving to FSI, CCI, and the number of SPC. Concerning FSI, three groups were established: Group 1 (from 44 to 60 days), Group 2 (from 60 to 70 days), and Group 3 (from 70 to 80 days). For CCI, two groups were considered: Group 1 (<110 days) and Group 2 (>110 days). Body condition score (BCS) was estimated the day of blood sampling on the basis of 1-5 scale [11].

### Blood sampling

Blood samples were obtained by coccygeal venipuncture with EDTA-anticoagulated vacutainer tubes. Tubes for plasma collection were rapidly cooled on crushed ice and transported to the laboratory. Blood samples were then centrifuged at 3000× *g* for 10 min, and the supernatant plasma was frozen at −20°C until analysis.

### Oxidative stress markers and biochemical parameters analysis

Oxidative stress parameters were measured by spectrophotometry (Jenway spectrophotometer, UK) and the biochemical parameters with AE-600 Biochemical analyzer, (Erma Inc. - Japan). Plasma was also analyzed for glucose (Glu), total lipids (Lip T), cholesterol (Chol), triglycerides, albumin, total proteins (Pro T), calcium (Cal), urea, creatinine (Crea), direct bilirubin DB), glutamic pyruvic transaminase (GPT), glutamic oxaloacetic transaminase (GOT), and alkaline phosphatase ALP).

### MDA concentration

Plasma MDA concentration (µmol/mL) was measured according to the method of Ohkawa *et al*. [12]. An aliquot of 100 µL was added to a reaction mixture containing 50 µL of 8.1% sodium dodecyl sulfate, 375 µL of 20.0% acetic acid (pH 3.5), 375 µl of 0.8% thiobarbituric acid. Samples were then boiled for 1 h at 95°C and centrifuged at 3000 g for 10 min. The absorbance of the supernatant was measured at 532 nm, and MDA content was expressed as µmol/ml (ε = 1.56 × 10^5^ mmol/L/cm).

### GST activity

The activity of GST was measured according to the method of Habig *et al*. [13]. The enzyme activity was expressed as nmol glutathione oxidation per minute at 25°C and was calculated using a molar extinction coefficient of 9.6 × 10^3^/M/c mat 340 nm wavelength.

### Statistical analysis

The results were analyzed using SPSS software for Windows (version 20.0; SPSS Inc., Chicago, IL, USA). The effect of reproduction traits and the BCS on MDA concentration and GST activity in the cows were analyzed using ANOVA. Differences were considered significant at p<0.05 and p<0.01. Shapiro–Wilk’s test was performed to examine whether variables were distributed normally. Correlation coefficients between MDA concentration and GST activity and biochemical parameters in plasma were calculated by Spearman’s method. Statistical significance of the correlation coefficients was tested at the level of p<0.05 and p<0.01.

## Results

### MDA plasma concentration and GST activity

#### Calving to FSI

**Figure-1:**
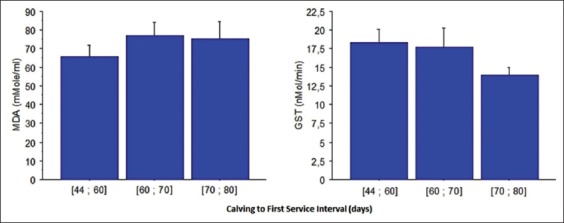
Plasma malondialdehyde concentration and glutathione S-transferase activity in cows according to calving to first service interval (mean±standard error).

MDA concentrations and GST activity according to FSI are shown in Figure-1. Both MDA and GST activity were not significantly different between the studied groups. Nevertheless, MDA tended to present lowest values in Group 1 (65.92±6.02 µmol/ml) corresponding to intervals from 44 to 60 days of FSI. For higher intervals, we observed more lipid peroxidation expressed with higher values of MDA.

GST showed inversely patterns with a tendency to present highest values in cows inseminated 44-60 days postpartum (18.34±1.76 nmol/mn in Group 1).

### CCI

**Figure-2:**
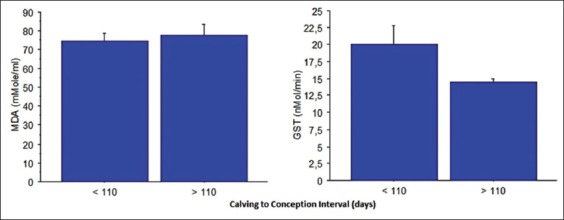
Plasma malondialdehyde concentration and glutathione S-transferase activity in cows according to calving to conception interval (mean±standard error).

MDA concentration and GST activity according to CCI are represented in Figure-2. Concerning MDA, close values were observed for cows conceiving before or after 110 days postpartum (74.36±4.01 vs. 77.67±5.71 µmol/ml). GST activity showed largely higher values in cows conceiving before 110 (19.99±2.79 nmol/mn) than those conceiving later (>110 days) with 14.38±0.61 nmol/mn, even if the difference did not achieve statistical significance (p=0.06).

### Number of SPC

**Figure-3:**
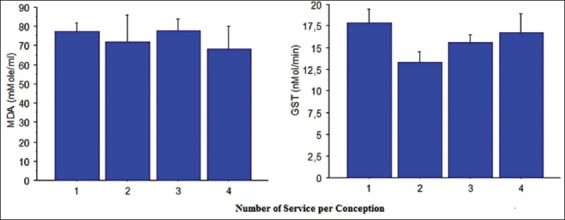
Plasma malondialdehyde concentration and glutathione S-transferase activity in cows according to service number (mean±standard error).

The number of services did not affect MDA concentration and GST activity (Figure-3) significantly. MDA concentrations fluctuated slightly between close values 68.16±12.11 and 77.58±5.87 µmol/ml. The highest values of GST activity (17.89±1.55 nmol/mn) were observed in cows conceiving at the first service.

### BCS

**Figure-4:**
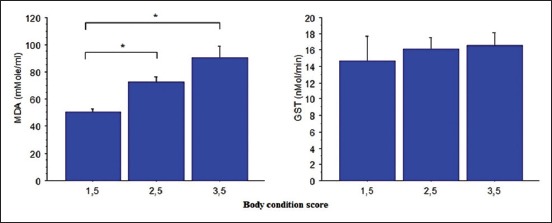
Plasma malondialdehyde concentration and glutathione S-transferase activity according to cows body condition score notes (mean±standard error).

MDA values and GST activity in each of the three BCS groups are presented in Figure-4. Cows with 1.5 of BCS showed the lowest values (50.0±3.03 µmol/ml) (p<0.05) compared to cows with 2.5 (72.58±3.54 µmol/ml) and 3.5 of BCS (90.32±8.62 µmol/ml) (p<0.05). Not significantly different was found between the groups with BCS notation 2.5 and 3.5. In another hand, despite the absence of statistical significance, GST activity showed a tendency to be elevated with the increased BCS. However, a tendency was to observe higher GST values with increased BSC with 14.71±3.0, 16.1±1.44, and 16.6±1.55 nmol/mn in 1.5, 2.5, and 3.5 BCS groups, respectively.

### Correlation between MDA concentration and GST activity with biochemical parameters

Correlations between MDA concentration and GST activity and biochemical parameters are shown in Table-1. No statistically significant correlations were found concerning GST. There were positive correlations between MDA and Glu (r=0.389; p<0.05), Glu and Cal (r=0.473; p<0.002), Lip T and Chol (r=0.343; p<0.05), Chol and D bilirubin (r=0.321; p<0.05), and Pro T and ALP (r=0.435; p<0.005). Significantly, negative correlations between MDA and Chol (r=−0.336; p<0.05), Chol and Crea (r=−0.445; p<0.006), urea and Bili D (r=−0.420; p<0.01) were observed (Table-1).

**Table-1:** Spearman correlation coefficients for MDA and GST activity and biochemical plasma parameters.

	MDA	GST	Glu	Lip T	Chol	Trigly	Albu	Prot T	Urea	Crea	GOT	GPT	ALP	DB	Cal
MDA	1	0.021	0.389*	−0.144	−0.336*	−0.087	0.178	−0.041	−0.094	0.021	0.095	−0.306	−0.046	−0.065	0.133
GST		1	0.092	0.204	0.171	−0.216	0.100	−0.066	−0.124	−0.315	0.198	−0.236	0.111	0.108	0.108
Glu			1	0.087	−0.176	−0.015	−0.075	−0.174	−0.111	−0.049	0.111	−0.192	−0.155	−0.087	0.473**
Lip T				1	0.343*	0.019	−0.070	0.011	−0.093	−0.215	−0.127	0.059	−0.022	0.288	0.184
Chol					1	−0.012	−0.028	−0.200	−0.026	−0.445**	0.191	0.012	−0.113	0.321*	0.056
Trig						1	0.285	−0.005	−0.143	0.195	0.052	0.212	−0.079	0.026	0.144
ALB							1	−0.290	−0.045	0.111	0.080	0.012	−0.244	0.102	−0.031
Prot T								1	−0.177	−0.083	−0.162	0.057	0.435**	−0.205	−0.107
Urea									1	−0.116	0.100	0.063	−0.183	−0.420**	0.120
Crea										1	−0.177	0.173	0.122	−0.074	−0.041
GOT											1	−0.129	−0.139	0.066	0.156
GPT												1	−0.109	−0.100	−0.151
ALP													1	−0.080	−0.042
DB														1	−0.156
Cal															1

* Indicate p<0.05 and

** indicate p<0.01. MDA=Malondialdehyde, GST=Glutathione S-transferase, Lip T=Total lipids, Chol=Cholesterol, Trig=Triglycerides, ALB=Albumin, Prot T=Total proteins, Crea=Creatinine, GOT=Glutamic oxaloacetic transaminase, GPT=Glutamic pyruvic transaminase, DB=Direct bilirubin, Cal=Calcium, ALP=alkaline phosphatase, Glu=Glucose

## Discussion

Different studies have indicated that increased production of ROS generating oxidative stress may contribute to peripartal metabolic and reproductive disorders and diseases [14,15] essentially through the decrease of enzymatic antioxidant activities [16]. In the current study, we investigate existing relationships between oxidative stress status, explored through MDA concentrations and GST activity on the one hand, and different reproductive success parameters on the other hand. We particularly considered intervals from CFS and CCI, parameters indicating resumption of estrus and ovulation and hence reproductive efficiency in dairy cows. MDA is one of the most major metabolites of lipids peroxidation and oxidative stress endpoint product that reveals essentially the impacts on cell membrane. GST, an antioxidant enzyme, is also considered as one of the keys biomolecules directly related to the extent of oxidative stress. These data support the findings of Turk *et al*. [14] and Colakoglu *et al*. [5] concerning MDA concentration measured during early puerperium and late puerperium until mid-lactation. All previous studies agree that significant changes in oxidative stress are observed, especially during the transition period [5,14,15,17]. In fact, increasing the amount of glutamine in the transition period has effective effects on increasing antioxidant capacity and can lead to increased dry matter intake [18].

Similarly, our results are in agreement with those reported by Celi *et al*. [19] who found that plasma concentration of reactive oxidant metabolites and biological antioxidant potential (BAP) is not related to the success of artificial insemination. Castro *et al*. [20] demonstrate that paraoxonase 1 (PON1) activity, as an inhibitor of oxidation of low-density lipoprotein and cell membranes, is not associated with follicle diameter and time of ovulation [20]. Furthermore, no difference in serum PON1 activity was observed between ovulating and non-ovulating cows. However, Talukder *et al*. [4] indicated a significant increase of BAP in an-ovulated cows at 48 h and 60 h of PGF_2α_ treatment. The authors hypothesized that PGF_2α_ treatment was unable to generate adequate amounts of free radicals in an-ovulated cows and consequently resulted to ovulation failure.

BCS remains a convenient informative tool in the management of cow breeding and nutrition. It provides information on the state of body reserves and their mobilization. On the other hand, lipid peroxidation is one of the most important expressions of oxidative stress induced by ROS. Therefore, MDA is the most frequently used biomarker indicating the extent of ROS-induced oxidative stress [16,19]. From MDA concentration, the present work showed that there was a significant difference among BCS groups. Cows with BCS=1.5 showed the lowest MDA concentration compared to cows with 2.5 and 3.5 of BCS. This finding is similar to those reported by Castillo *et al*. [21] and O’Boyle *et al*. [22] in late-lactation cows suggesting that the ROS levels may not be sufficient to cause increased lipid peroxidation.

In our study, there were no significant differences in MDA concentration and GST activity according to different reproductive parameters. In fact, no significant correlation was observed between MDA and GST independently of the studied group. Similarly, no significant correlation was observed between GST and blood biochemical parameters. In contrast, MDA showed, on the one hand, positive correlation with Glu and on the other hand negative correlation with Chol. This is rationally understandable; as more lipid oxidation is important, less lipid substrates are abundant. In fact, metabolic status is a reflection of the whole physiological interactions involving simultaneously different biomolecules [21].

## Conclusion

The results of the present study revealed no significant effect of oxidative stress status on dairy cattle reproductive performances. However, the results revealed a tendency to observe relatively exacerbated oxidative stress in cows with altered reproduction performances. The current results reinforce the contradictory debate concerning the existing relationships between oxidative stress and reproduction performances.

## Authors’ Contributions

MI and SB have conceived and designed the study. SB was responsible for sample collection and the manuscript writing under the guidance of MI. ST, LA, SoT, and SB worked out a major part of the analysis protocols and carried out the laboratory examinations. All authors read and approved the final manuscript.
